# Attributes of High-Performance Electron Transport Layers for Perovskite Solar Cells on Flexible PET versus on Glass

**DOI:** 10.1021/acsaem.1c03311

**Published:** 2022-04-06

**Authors:** Marwa Dkhili, Giulia Lucarelli, Francesca De Rossi, Babak Taheri, Khadija Hammedi, Hatem Ezzaouia, Francesca Brunetti, Thomas M. Brown

**Affiliations:** † CHOSE (Centre for Hybrid and Organic Solar Energy), Department of Electronic Engineering, University of Rome Tor Vergata, Via del Politecnico 1, 00133 Rome, Italy; ‡ Laboratory of Semiconductors, Nanostructures and Advanced Technology (LSNTA), Research and Technology Centre of Energy (CRTEn), BP 95, 2050 Hammam-Lif, Tunisia; § Photovoltaic Laboratory, Research and Technology Centre of Energy (CRTEn), BP 95, 2050 Hammam-Lif, Tunisia; ∥ Faculty of Sciences of Tunis, El Manar University, 2092 Tunis, Tunisia

**Keywords:** flexible perovskite solar cells, substrates, rigid perovskite solar cells, solution-processed electron transport layer, SnO_2_ layer, ZnO/SnO_2_ double layer, flexible versus rigid, polyethylene terephthalate

## Abstract

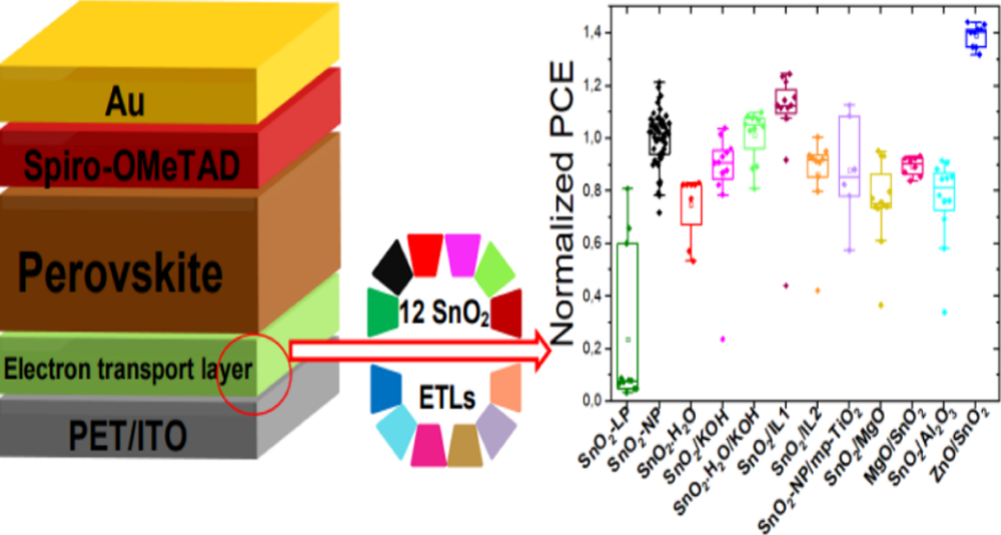

Electron transport layers (ETLs) play a fundamental role in perovskite solar cells (PSCs) through charge extraction. Here, we developed flexible PSCs on 12 different kinds of ETLs based on SnO_2_. We show that ETLs need to be specifically developed for plastic substrates in order to attain 15% efficient flexible cells. Recipes developed for glass substrates do not typically transfer directly. Among all the ETLs, ZnO/SnO_2_ double layers delivered the highest average power conversion efficiency of 14.6% (best cell 14.8%), 39% higher than that of flexible cells of the same batch based on SnO_2_-only ETLs. However, the cells with a single ETL made of SnO_2_ nanoparticles were found to be more stable as well as more efficient and reproducible than SnO_2_ formed from a liquid precursor (SnO_2_-LP). We aimed at increasing the understanding of what makes a good ETL on polyethylene terephthalate (PET) substrates. More so than ensuring electron transport (as seen from on-current and series resistance analysis), delivering high shunt resistances (*R*_SH_) and lower recombination currents (*I*_off_) is key to obtain high efficiency. In fact, *R*_SH_ of PSCs fabricated on glass was twice as large, and *I*_off_ was 76% lower in relative terms, on average, than those on PET, indicating considerably better blocking behavior of ETLs on glass, which to a large extent explains the differences in average PCE (+29% in relative terms for glass vs PET) between these two types of devices. Importantly, we also found a clear trend for all ETLs and for different substrates between the wetting behavior of each surface and the final performance of the device, with efficiencies increasing with lower contact angles (ranging between ∼50 and 80°). Better wetting, with average contact angles being lower by 25% on glass versus PET, was conducive to delivering higher-quality layers and interfaces. This cognizance can help further optimize flexible devices and close the efficiency gap that still exists with their glass counterparts.

## 1 Introduction

Perovskite solar cells (PSCs) have evoked tremendous interest in solar energy researchers. PSCs combine high-efficiency and high-throughput manufacturing via solution processing.^1^ Whereas the main substrate has largely been glass, development of PSCs on flexible substrates has recently accelerated. This is because they are light, flexible, and processed at low-temperatures at very low cost,^2^ even via roll-to-roll manufacturing,^3^ and can be easily integrated in many objects conformally for both outdoor and indoor operation.^4−7^

In general, the photoactive perovskite layer (i) is sandwiched between an (n) electron transport layer (ETL) and a (p) hole transport layer (HTL). The ETL, in n–i–p architectures, plays a vital role in achieving high-performance solar cells: it promotes the collection of photogenerated electrons from the perovskite layer to the bottom electrode as well as suppressing recombination.^8^ The main ETLs for n–i–p architectures are largely consisted of metal oxides such as TiO_2_,^9^ ZnO,^10,11^ and SnO_2_.^12,13^

For flexible PSCs (FPSCs), deposition of thin-film ETLs must be carried out at low temperatures, that is, below 150 °C.^14^ Wang et al. developed room-temperature-processed WO_x_ as ETL achieving a power conversion efficiency (PCE) of 15.85%.^15^ By introducing SnS_2_ as ETL, Chu et al. reported a power conversion efficiency of 13.2% with a negligible hysteresis effect in FPSCs.^16^ Low-temperature-processed Zn_2_SnO_4_ nanoparticles (NPs) have also been shown to be a good ETL for efficient FPSCs.^17−19^ Liu and Kelly demonstrated a PCE with 10% using ZnO NP as ETL.^20^ However, ZnO is found to accelerate the thermal degradation of perovskite films.^21,22^

SnO_2_ has become in recent years the prime candidate as ETL in FPSCs since it can be deposited and processed at low temperatures on plastic substrates,^23,24^ even though its performance in some reports can still suffer from large densities of surface trap states, resulting in photocurrent hysteresis and poor fill factors (FFs).^25,26^ In fact, SnO_2_ can be deposited via different techniques, from sputtering to solution processing of precursor solutions or NP dispersions.^27^ There have been many reports on PSCs, mainly on rigid glass substrates, which applied interface treatment to SnO_2_ with materials such as polyelectrolytes,^28^ H_2_O,^29^ and KOH^30^ or proposed the addition of a second layer of metal oxide either over or underneath SnO_2_. For example, solid-state ionic liquids (SS-ILs) have been applied to function as independent ETLs^31,32^ or used to modify another ETL such as TiO_2_.^31^ Bu et al. have introduced potassium treatment as a passivation strategy for SnO_2_ ETLs in order to suppress the hysteresis effect and the surface defects of the perovskite.^30^ By introducing a Zn_*X*_Sn_*X*_O/SnO_2_ bilayer ETL, Thambidurai et al. demonstrated an efficiency of 19% on rigid PSCs.^33^ The ZnS interlayer improved the efficiency and stability of PSCs via passivation of the ZnO surface and reduction of interfacial charge recombination.^34^ Chung et al. achieved record efficiency on flexible cells using a porous-planar structure as an ETL.^35^ A ZnO–SnO_2_-cascaded ETL was employed to improve the interface stability and efficiency of PSCs.^36^ Dagar et al. developed all-solution-processed ETLs: SnO_2_/meso-TiO_2_ bilayers on flexible substrates and SnO_2_/MgO, SnO_2_/Al_2_O_3_ bilayers on rigid substrates, improving PCEs significantly^7,37,38^ by enhancing wettability, rectification ratios, and shunt resistances.^37,38^ The ETL overlayer has also been shown to reduce the work function of the composite electrode thus helping improve electron extraction.^39^

Most of these works have been carried out on glass substrates and have not been investigated on flexible polyethylene terephthalate/indium tin oxide (PET/ITO) substrates. The morphological, chemical, and/or wetting properties of the PET/ITO substrates can be very different from those of glass.^28,40,41^ Thus, the quality and performance of ETLs will generally differ when transferring recipes for glass substrates to flexible substrates. Here, we carried out a systematic investigation of many different strategies and materials for SnO_2_-based ETLs, which can be summarized as follows: single-layer SnO_2_, both from NP dispersion (SnO_2_-NP) and from liquid precursors (SnO_2_-LPs); single-layer SnO_2_ with H_2_O and KOH surface treatments; and double-layer ETLs, that is, SnO_2_/SS-IL, SnO_2_/mp-TiO_2_, SnO_2_/MgO, MgO/SnO_2_, SnO_2_/Al_2_O_3_, and ZnO/SnO_2_. Whereas some strategies worked on glass, improving the PCE compared to the control devices, as described in the literature, there were no significant enhancements when transferred to flexible substrates. We instead found that double layers of ZnO/SnO_2_ led to a significant enhancement in efficiency in flexible cells. Furthermore, SnO_2_ NPs worked considerably better and more reproducibly compared to SnO_2_ grown from a liquid precursor. SnO_2_-NP ETLs also led to the more stable FPSCs. Importantly, the systematic investigation on so many different cells and cell types differing only by the type of ETL enabled us to gain a deeper understanding of what makes a good ETL for FPSCs and the differences compared to those manufactured on glass, relating to parameters such as series and shunt resistances, recombination, injection currents, and wettability, which limit the efficiency achievable on this type of more challenging substrate. Quantifiable differences were found for these parameters in order to achieve high-efficiency ETLs for FPSCs.

## 2 Experimental Section

### 2.1 Materials

Zinc oxide dispersion, tin chloride (SnCl_2_·2H_2_O) dehydrate, Al_2_O_3_ (aluminum oxide) NPs with <50 nm particle size, magnesium acetate tetrahydrate [(CH_3_COO)2Mg·4H_2_O], lead(II) bromide (PbBr_2_), cesium iodide (CsI), *tert*-butylpyridine (TBP), Li bis(trifluoromethanesulfonyl)imide (Li-TFSI), potassium hydroxide solution (KOH), and solvents dimethyl sulfoxide (DMSO anhydrous, ≥99.9%), *N*,*N*-dimethylformamide (DMF anhydrous, 99.8%), diethyl ether (99.0%), ethanol (99.8%), and 2-propanol anhydrous 99.5% were purchased from Sigma-Aldrich. Tin(IV) oxide 15% in H_2_O colloidal dispersion and 1-benzyl-3-methylimidazolium chloride (IL1) were purchased from Alpha Aesar. 1-Butyl-3-methylimidazolium tetrafluoroborate (IL2) was purchased from Acros Organics. Lead(II) iodide (PbI_2_) (99.99%, trace metal basis) was purchased from TCI Deutschland GmbH. Formamidinium iodide (FAI) methylammonium bromide (MABr), and methylammonium iodide (MAI) were purchased from GreatCell Solar. 18NR-T titania paste and cobalt salt (III) FK209 TFSI (98%) were purchased from Dyesol Limited. 2,2′,7,7′-Tetrakis-(*N*,*N*-di-*p*-methoxyphenylamine)-9,9′-spirobifluorene (spiro-OMeTAD) (≥99.8%) was purchased from Borun New Material Technology Co., Ltd.

### 2.2 Device Fabrication

PET/ITO (125 μm thickness, 15 Ω sq^–1^ Flexvue) and glass/ITO (Kintec 8 Ω sq^–1^) were cut mechanically to obtain 2.5 × 2.5 cm substrates. The ITO electrode was patterned using a CO_2_ laser and raster scanning laser. The substrates were cleaned in water and soap, isopropanol, and water for 10 min each using an ultrasonic bath, followed by drying with compressed air. The architecture used for the fabrication of our FPSCs was PET/ITO/ETL/perovskite/spiro-OMeTAD/Au and glass/ITO/ETL/perovskite/spiro-OMeTAD/Au for our rigid ones. To enhance the wettability of the ITO electrode, the substrates were treated with UV irradiation for 10 min. A ZnO ETL was deposited on the PET/ITO substrate by a spin coating process (3000 rpm, 30 s) using ZnO NP dispersion in ethanol (Sigma-Aldrich, 40 wt %) which was further diluted with ethanol in order to obtain 4 wt % concentration. The ZnO film was annealed at 100 °C for 1 h, followed by a UV irradiation treatment for 10 min. Afterward, SnO_2_ ETL (15% in H_2_O colloidal dispersion, Alfa Aesar) was spin coated over the ZnO layer at a spin speed of 6000 rpm for 35 s obtaining a ∼60 nm thick ZnO/SnO_2_ bilayer, with a ∼40 nm thick SnO_2_ layer. SnO_2_ was also annealed at 100 °C for 1 h and further exposed to a UV irradiation treatment for 10 min. For SnO_2_-LP, the ETL was deposited by spin coating a 0.1 M precursor solution of SnCl_2_/2H_2_O in ethanol at 1500 rpm for 30 s, followed by a second step at 2500 rpm for 30 s; films were annealed at 150 °C for 1 h. For the SnO_2_-NP ETL modified by H_2_O and KOH interface treatments, the ETL was first dipped in H_2_O at 90 °C for 1 h (SnO_2_–H_2_O), the second treatment was the spin coating of 0.01 M KOH over the SnO_2_ layer (SnO_2_/KOH), and finally the last treatment was the combination of the previous two treatments, that is, dipping in H_2_O at 90 °C for 1 h and then spin coating of 0.01 M KOH (SnO_2_–H_2_O/KOH). The ionic-liquid ETLs were deposited by spin coating a 0.3 wt % of IL1 in isopropanol and a 0.3 wt % of IL2 in methanol at 5000 rpm for 60 s and 4000 rpm for 60 s, respectively. TiO_2_, MgO, and Al_2_O_3_ double-layer ETLs were deposited following the optimized procedure reported previously by Dagar et al.^7,37,38^

The triple cation perovskite solution was prepared by dissolving PbI_2_, FAI, PbBr_2_, MABr, and CsI in DMF/DMSO mixed solvents (7:6:2:4 v/v). After stirring overnight at room temperature, the perovskite solution was spin coated by means of a two-step process at 1000 rpm for 10 s with five acceleration steps, followed by 5000 rpm for 30 s with two acceleration steps. During the second step, 150 μL of chlorobenzene was poured on the spinning substrate 7 s prior to the end of the process. The perovskite films were annealed at 100° C for 1 h inside a nitrogen-filled glovebox. In case of cells based on SnO_2_-LP ETL, the perovskite layer was MAPI as this type of perovskite worked better with SnO_2_-LP.^7^ The MAPbI_3_ solution was prepared by dissolving PbI_2_ and MAI in a 1:1 molar ratio in a solvent mixture composed of DMF/DMSO (9:1, v/v) to obtain a final concentration of 1.4 M. The same spin-coating process of triple cation perovskite was used, but instead of CB, 0.7 mL of diethyl ether was dropped on the rotating substrate. Perovskite films were annealed at 50 °C for 2 min and 100 °C for 10 min; the process was carried out in air.

For the deposition of the HTL, spiro-OMeTAD was dissolved in chlorobenzene at a concentration of 73.5 mg/mL and doped with TBP (26.77 μL/mL), Li-TFSI (16.6 μL/mL of a 520 mg/mL solution in acetonitrile), and cobalt(III) (7.2 μL/mL of a 376 mg/mL solution in acetonitrile). The spiro-OMeTAD solution was spin coated over the perovskite layer at 2000 rpm for 20 s. The thickness of the triple cation perovskite and MAPI perovskites layers was ∼600 nm. The thickness of the HTL was ∼250 nm as measured with a profilometer. Finally, 90 nm gold contacts were thermally evaporated under high vacuum (below 10–6 mbar) through a shadow mask with a 0.16 cm^2^ area.

### 2.3 Device Measurements

The electrical measurements at 1 sun (AM1.5G, 100 mW/cm^2^, 25 °C) were performed at by means of a Keithley 2420 source meter under an ABET sun 2000 solar simulator class A as the light source. During the measurements, the devices were masked with a black tape with a 0.09 cm^2^ aperture. Dark *J*–*V* measurements were carried out with a modular testing platform (Arkeo 4 channel *J*–*V* system-Cicci Research s.r.l.) consisting of a high-speed source meter unit (600 K samples/s). For the stability measurements, unencapsulated devices were tested under white light-emitting diodes (LEDs) (4200 K) at 1 equivalent sun under ambient conditions. The MPP was measured via perturb and observed algorithm implemented onto a commercial apparatus (Arkeo-Ariadne, Cicci Research s.r.l.) based on a set of four-wire independent source meters. Energy-dispersive X-ray (EDX) and scanning electron microscopic (SEM) images were captured with a scanning electron microscope (Leo Supra 35) equipped with an INCAx-Sight Oxford Instruments X-EDS.

## 3 Results and Discussion

We first present a systematic study on flexible substrates that includes 12 different ETL types on PET/ITO and over 160 cells. We will subsequently analyze the more interesting four types of ETLs and carry out, over one simultaneous experiment, a vis-a-vis comparison between devices manufactured on PET and on glass in order to understand and quantify differences between the two types of devices (glass and PET).

### 3.1 12 Different ETLs for FPSCs

We fabricated 12 different sets of PSCs on PET/ITO substrates; each with a distinct ETL based on SnO_2_ as the common material. All incorporate a compact SnO_2_ ETL deposited by spin coating, some with the addition of another metal oxide and others with a polyelectrolyte or an interface treatment of the SnO_2_ layer prior to the deposition of the overlaying perovskite film. Stacks were completed with a perovskite active layer, spiro-OMeTAD HTL, and an Au top electrode. All layers except the evaporated metal contact were deposited by solution-processing at low temperatures (<150 °C).

**Figure 1:**
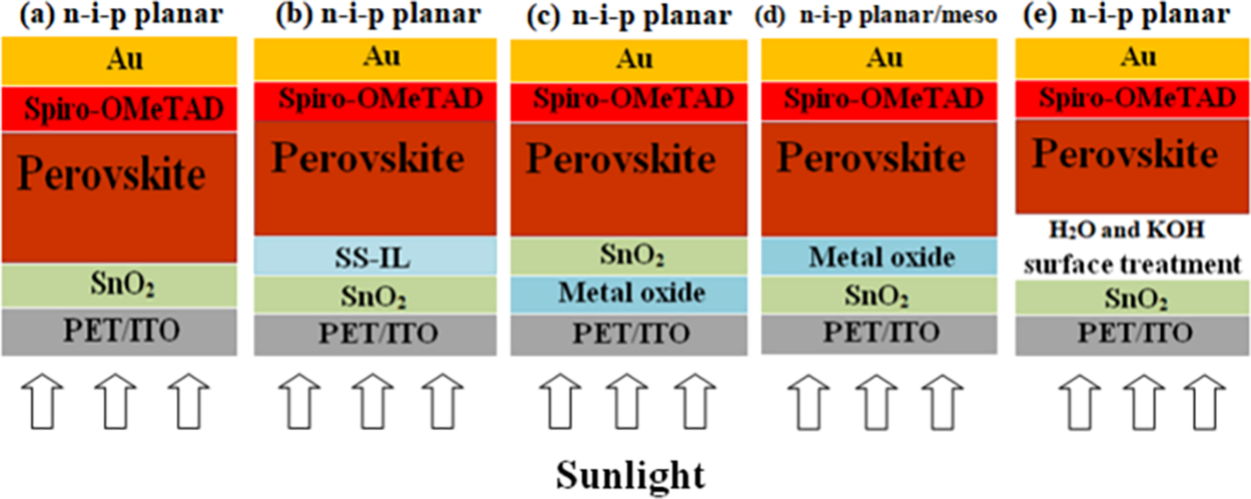
Schematic architectures of the FPSC devices investigated.

As schematized in Figure 1, there were five different architectures of FPSCs in 12 different material configurations in total that we investigated. The first type (Figure 1a) consisted in PSCs with SnO_2_ only as ETL. Two different SnO_2_ ETLs were tested, one based on a liquid precursor (SnO_2_-LP), deposited by spin coating a SnCl_2_ solution in ethanol, and the other consisting in a layer of ready-made NPs (SnO_2_-NP), deposited by spin coating a water-based colloidal dispersion. The second type of configuration (Figure 1b) contained SnO_2_/SS-LL bilayer ETLs. We tested two types of SS-IL, that is, IL1^31^ and IL2,^42^ which were spin coated over SnO_2_. A third configuration (Figure 1c) consisted in bilayers with a second metal oxide layer, that is, ZnO or MgO, deposited on the PET/ITO substrate below the SnO_2_ ETL. In contrast, in the fourth type of configuration (Figure 1d), the metal oxide layer, that is, MgO, Al_2_O_3_, or mesoporous TiO_2_, was deposited over the SnO_2_ layer. In the last configuration (Figure 1e), the SnO_2_ ETL was subjected to three types of interface treatment using H_2_O and KOH: dipping in H_2_O (SnO_2_–H_2_O), spin coating of KOH over the SnO_2_ layer (SnO_2_/KOH), and a combination of the previous two treatments (dipping in water followed by KOH treatment, labeled as SnO_2_–H_2_O/KOH). Summarizing, the 12 different ETLs investigated were SnO_2_-LP, SnO_2_-NP, SnO_2_–H_2_O, SnO_2_/KOH, SnO_2_–H_2_O/KOH, SnO_2_/IL1, SnO_2_/IL2, SnO_2_/mp-TiO_2_, SnO_2_/MgO, MgO/SnO_2_, SnO_2_/Al_2_O_3_, and ZnO/SnO_2_. For the composite ETLs (configurations b, c, d, and e), the SnO_2_ ETLs were made out of NPs (i.e., SnO_2_-NP) since this led to better performance. The symbol “NP” will not be repeated when describing the SnO_2_-NP for the sake of simplicity.

#### 3.1.1 Efficiency of FPSCs versus ETLs

**Figure 2:**
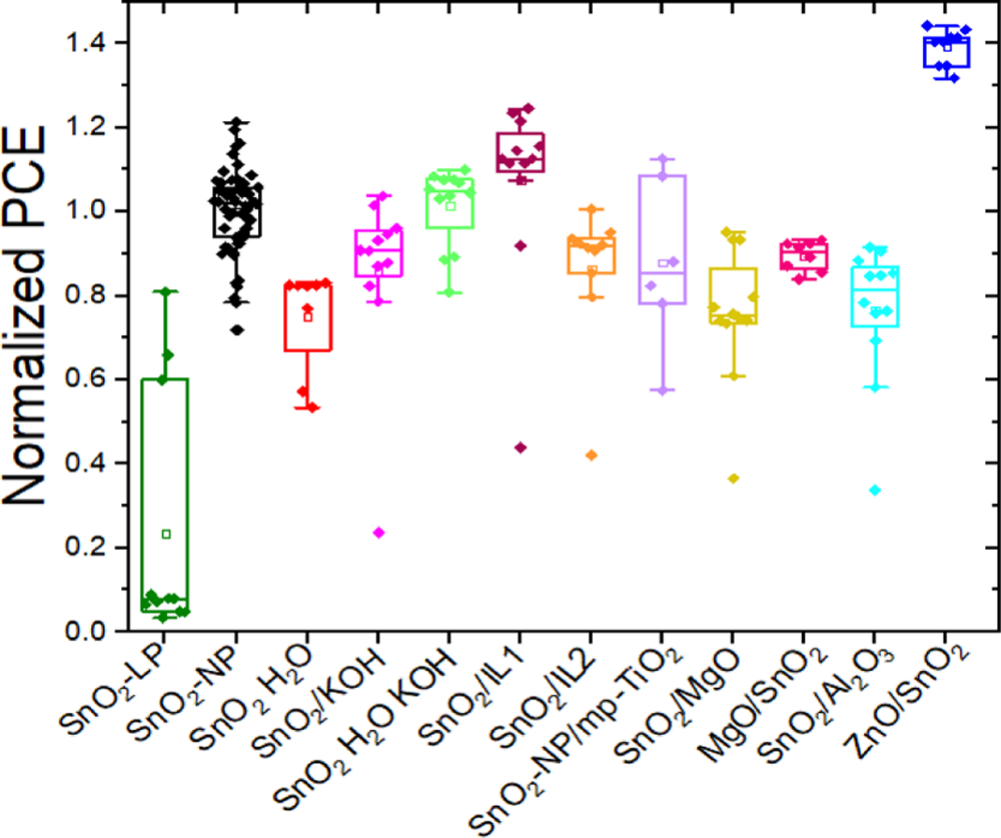
Statistical distribution of PCEs of FPSCs on PET/ITO substrates based on 12 different ETLs, that is, SnO_2_-LP, SnO_2_-NP, SnO_2_–H_2_O, SnO_2_/KOH, SnO_2_–H_2_O/KOH, SnO_2_/IL1, SnO_2_/IL2, SnO_2_/mp-TiO_2_, SnO_2_/MgO, MgO/SnO_2_, SnO_2_/Al_2_O_3_, and ZnO/SnO_2_, where IL1 and IL2 are 1-benzyl-3-methylimidazolium chloride and 1-butyl-3-methylimidazolium tetrafluoroborate, respectively. PCE values were normalized by the average efficiency of the reference cell of each batch.

Figure 2 presents the PCEs of the flexible cells fabricated with the 12 different ETLs described above. Different cell configurations were developed over the course of months. Each time, cells with SnO_2_-NP ETLs were fabricated and used as reference devices. The PCEs of the other configurations are normalized to the average PCE of the reference SnO_2_-NP cells each time to take into account fluctuations in performance that can occur from batch to batch and over different periods of time (which can be significant). The statistical distribution of the non-normalized PCEs of the 12 different configurations carried out in different batches is reported in Figure S1. Both figures show that ETLs do have a considerable bearing on the power output of the solar cells. When switching from SnO_2_-LP to SnO_2_-NP, the average PCE increases by 250%. Not only a boost in performance was observed but the reproducibility also increased dramatically. In fact, the standard deviation was 4.35% in absolute value (143% in relative terms) in the former case but narrowed to 2.2% (down to 23% in relative terms) for SnO_2_-NP. Thus, the deposition of a SnO_2_-NP compact layer is much more consistent over different cells and different batches (59 cells were tested for the SnO_2_-NP case) compared to the liquid precursor formulation. Whereas in glass-based devices, metal oxide bilayers comprising MgO or Al_2_O_3_ gave significant boost in efficiency,^37,38^ this was not the case consistently with flexible devices. Similarly, with the ILs, the boost is not statistically significant for the flexible cells. The only case where a significant improvement in efficiency (39% in relative terms) was observed with respect to the SnO_2_-NP single layer is represented by the ZnO/SnO_2_ bilayer.

#### 3.1.2 Flexible Cells with SnO_2_-NP and ZnO/SnO_2_ ETLs

**Figure 3:**
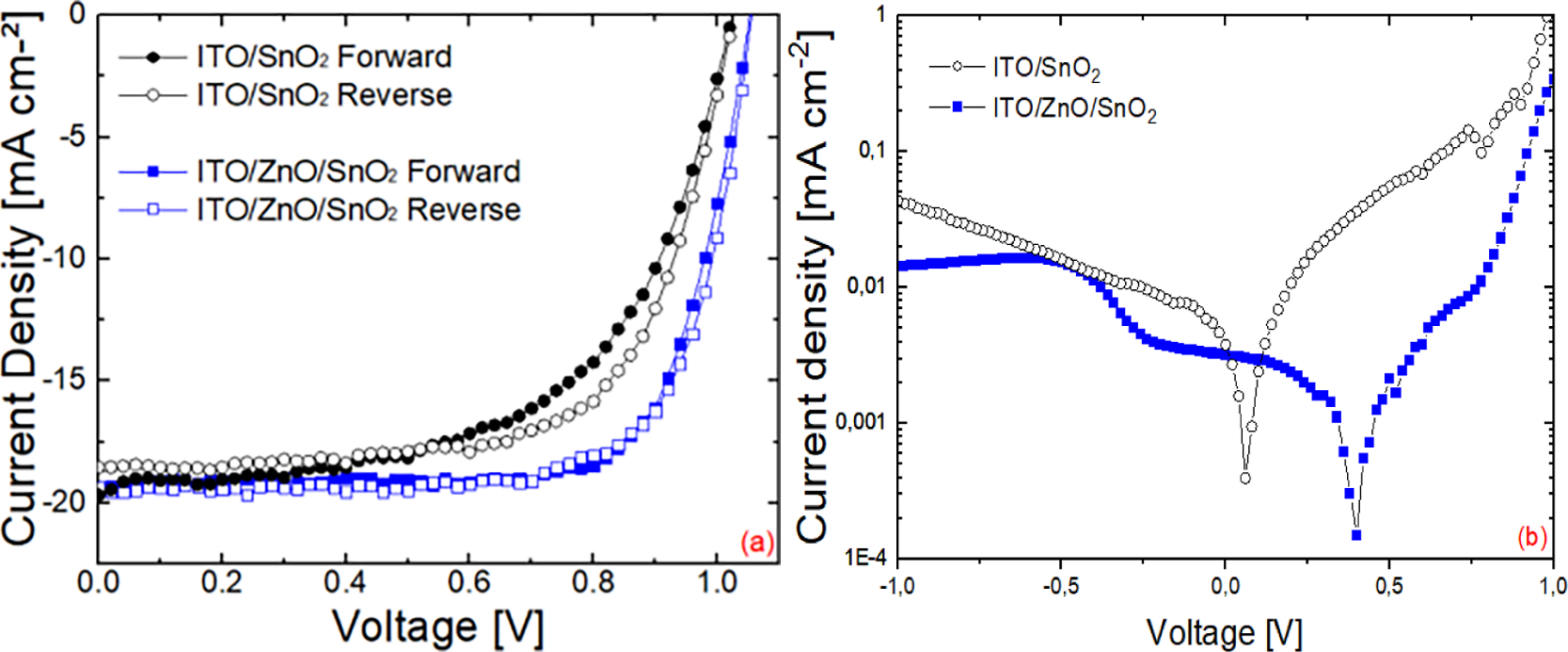
(a) *J*–*V* curves of champion PSCs with PET/ITO/ETL/perovskite/spiro-OMeTAD/Au architecture based on SnO_2_ (black circles) and ZnO/SnO_2_ (blue squared data points) ETLs under AM1.5G, 1000 W/m^2^ irradiation, measured in both forward and reverse scans. (b) Dark *J*–*V* characteristics of SnO_2_ and ZnO/SnO_2_-based PSCs.

The current density–voltage (*J*–*V*) characteristics of the best-performance FPSCs based on SnO_2_ and ZnO/SnO_2_ ETLs fabricated simultaneously in the same batch under one sun illumination are shown in Figure 3a. Table 1 lists the average photovoltaic (PV) parameters of the cells at one sun, including short-circuit current (*J*_SC_), open-circuit voltage (*V*_OC_), fill factor (FF), and PCE. For this particular batch, the flexible devices with a neat layer of SnO_2_-NP exhibit an average PCE of 10.5%, while the devices with a ZnO/SnO_2_ bilayer yield an average PCE of 14.6%. Thus, the insertion of the ZnO layer between the ITO cathode and the SnO_2_ layer was beneficial for the solar cell power output.

**Table 1:** PV Parameters, Open-Circuit Voltage (V_OC_), Short-Circuit Current Density (*J*_SC_), Fill Factor (FF), and Power Conversion Efficiency (PCE) of FPSCs Based on SnO_2_ and ZnO/SnO_2_ ETL Fabricated in the Same Batch

ETL	scan direction	*V*_OC_ [V]	*J*_SC_ [mA/cm^2^]	FF [ %]	PCE [ %]	PCE best cells [%]
SnO_2_	forward	1.03 ± 0.01	19.59 ± 0.27	48.23 ± 9.28	9.79 ± 1.91	11.4
	reverse	1.038 ± 0.008	19.25 ± 0.66	52.6 ± 12.83	10.47 ± 2.19	12.7
ZnO/SnO_2_	forward	1.06 ± 0.01	19.41 ± 0.29	69.05 ± 3.81	14.23 ± 0.64	14.9
	reverse	1.063 ± 0.007	19.14 ± 0.3	71.45 ± 2.07	14.58 ± 0.51	14.8

**Figure 4:**
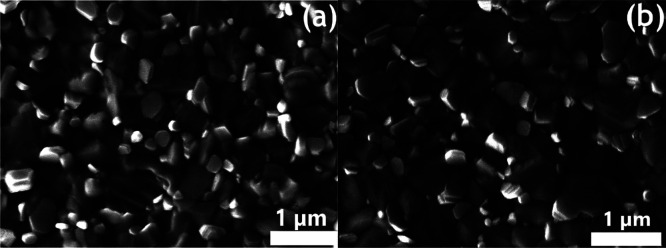
Top-view SEM images of perovskite films deposited on PET/ITO/SnO_2_ (a) and PET/ITO/ZnO/SnO_2_ (b).

The improvement in PCE is mainly attributed to the significant increase in FF and secondly to a smaller increase in V_OC_. Figure 3b displays the dark *J*–*V* characteristics of the best-performing PSCs with and without the ZnO interlayer. The ZnO/SnO_2_-based cells delivered smaller reverse current showing better blocking behavior and lower defect densities.^43,44^ Composite ETLs, in fact, as in this case, reduce both pinholes and surface trap states which can be present at some of the interfaces of single ETLs^34,37−39^ in addition to the reduction of charge recombination at interfaces,^45^ suggested by higher shunt resistance compared to the cells with SnO_2_ only.^38,46−48^ SEM images of the perovskite films deposited on SnO_2_ and ZnO/SnO_2_ ETLs (Figure 4) reveal that also the bulk perovskite film improves when growing over the composite ETL. In fact, the average grain size of the perovskite film grown on ITO/ZnO/SnO_2_ (Figure 4b) was 300–350 nm, larger than the ∼220 nm size for films grown on ITO/SnO_2_ (Figure 4a). This difference contributes to the improvement in FF and V_OC_, as well as in the hysteresis behavior.^49−52^

#### 3.1.3 Stability of Flexible Cells with Different ETLs

**Figure 5:**
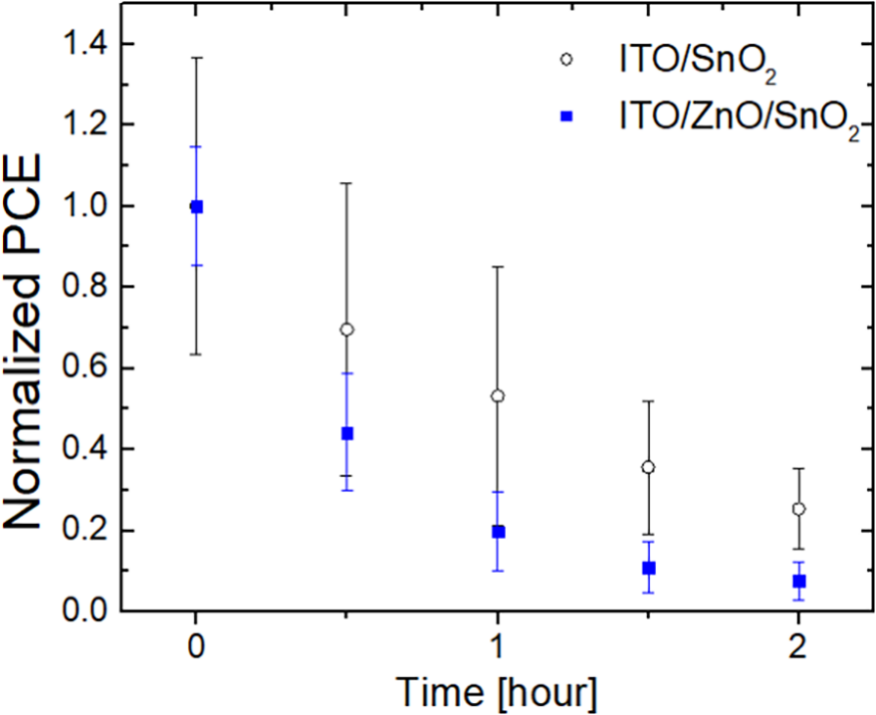
Light-soaking stability measurements at one sun illumination, under ambient temperature and RH in the range of 30–60%, over time of FPSCs with either SnO_2_ or ZnO/SnO_2_ ETLs.

We performed light-soaking stability tests on the cells based on SnO_2_-NP single ETL and with ZnO/SnO_2_-NP bilayer ETL. Unencapsulated cells were illuminated with a solar simulator at one sun equivalent irradiation from white LEDs over a period of 2 h while tracking their maximum power point and measured every 30 min under ambient conditions, that is, temperature (25 °C) and relative humidity (RH) (30–60% range), that is, according to the ISOS-L-1 stability protocol.^53^Figure 5 shows the light-soaking effect on the PCE of our devices. The cells with SnO_2_ only were found to be more stable than cells with the ZnO/SnO_2_ ETLs.

Although the PCE improved for fresh cells compared to the reference devices when depositing ZnO under SnO_2_, the latter did not completely avoid interaction with the underlying ZnO and thus degradation of the perovskite films.^21,22^ This is not surprising since the SnO_2_-NP film is made of individual NPs and so a completely compact film that avoids all contact between ZnO and the perovskite film is unattainable for layers that are as thin as these (i.e., <80 nm).

**Figure 6:**
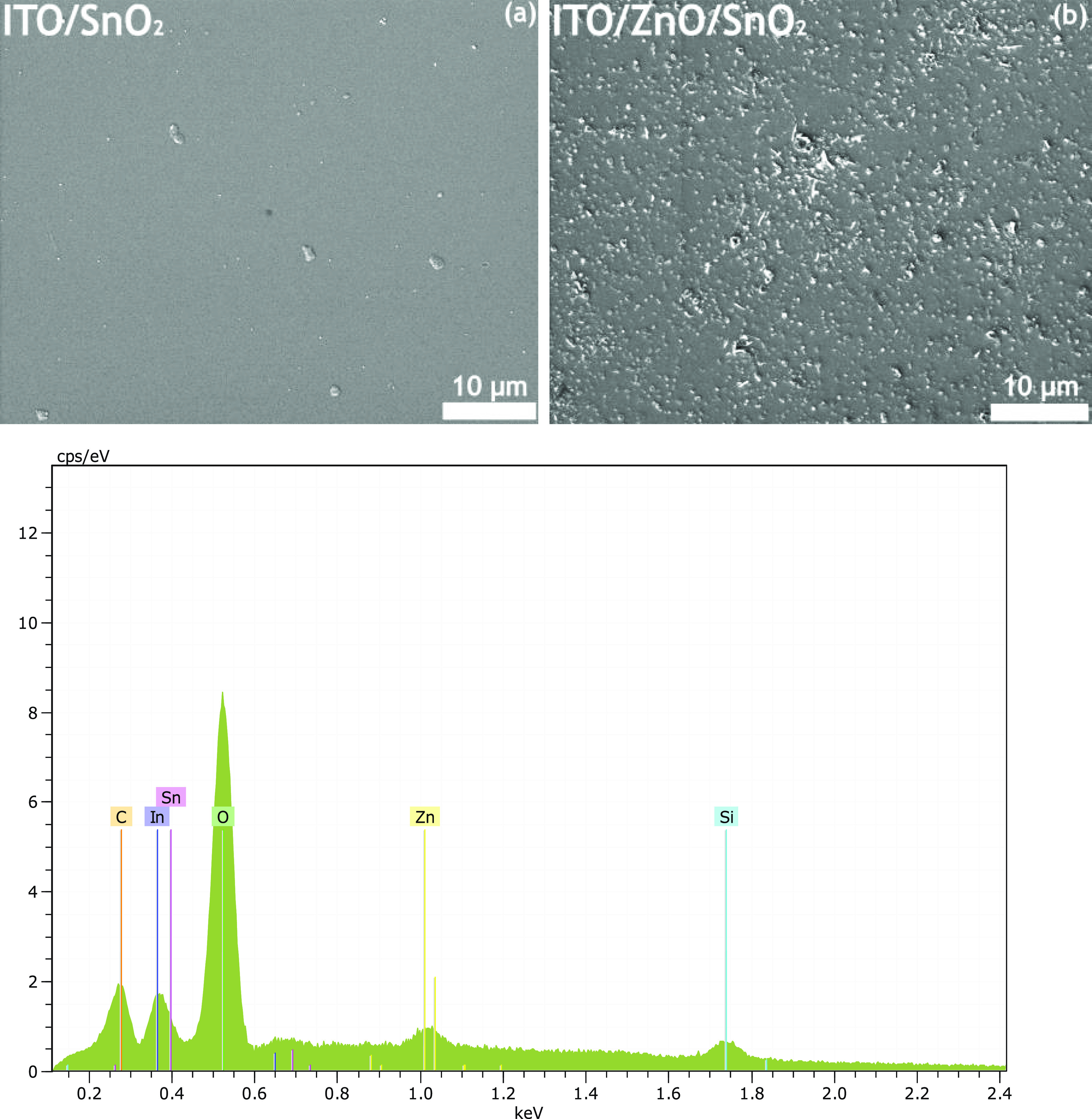
Top-view SEM images of (a) PET/ITO/SnO_2_, (b) PET/ITO/ZnO/SnO_2_ and EDX analysis of the PET/ITO/ZnO/SnO_2_ multilayer.

SEM images (Figure 6) show that the surface of ZnO/SnO_2_ was rougher than that of the SnO_2_-NPs, which can be attributed to the granular NPs of the ZnO layer formed after annealing.^54^ This may also explain the different morphologies of the perovskite layers growing over the two types of ETL. Notably, a large concentration of small holes/craters can be seen in the ZnO/SnO_2_ SEM. These are likely areas where the perovskite comes into contact with the underlying ZnO. EDX analysis, shown in Figure 6, reveals that the surface of the ZnO/SnO_2_ bilayer consists of C, In, Sn, O, ZnO, and Si at 9.22, 7.59, 1.03, 71.29, 1.60, and 9.27 at. %, respectively, confirming that the SnO_2_ overlayer did not completely avoid direct interaction of the perovskite films with the underlying ZnO explaining the poorer stability of the cell with this type of ETL.

We also performed light-soaking stability measurements on the batches with SnO_2_/MgO- and SnO_2_/Al_2_O_3_-based cells. As shown in Figures S2 and S3, cells with SnO_2_-NP ETLs display better stability compared to those with double-layer ETLs, that is, SnO_2_/MgO and SnO_2_/Al_2_O_3_. This is different to the previously reported case on glass substrates, where the double-layer strategy did lead to better stability with respect to the SnO_2_ single layer only.^37,46^ It is worth noting that the double- and single-layer ETLs developed on glass substrates in refs (37) and (38) were made with SnO_2_-LP, not with NP.

Regarding mechanical stability under bending, this is limited not by the perovskite or transport layers but by the flexibility of the ITO which has a safe bending radius of 7 mm, as shown in previous studies.^4,55,56^ New transparent electrodes would need to be designed for going down to very low curvatures.^56^

#### 3.1.4 SnO_2_-NP as the ETL of Choice for FPSCs

Our study shows that materials and recipes for PSCs on flexible substrates must be specifically developed and optimized for this particular substrate rather than just transferring them from glass-based devices since performance behavior will not match. Thus, if one wants to make highly efficient champion cells on flexible PET/ITO substrates, one could choose the ZnO/SnO_2_ ETL. However, considering stability together with more simple fabrication, then the better choice for ETL becomes that of SnO_2_-NP only. This NP-based SnO_2_-NP led to much more efficient and reproducible solar cells compared to the liquid precursor (SnO_2_-LP) alternative.

**Figure 7:**
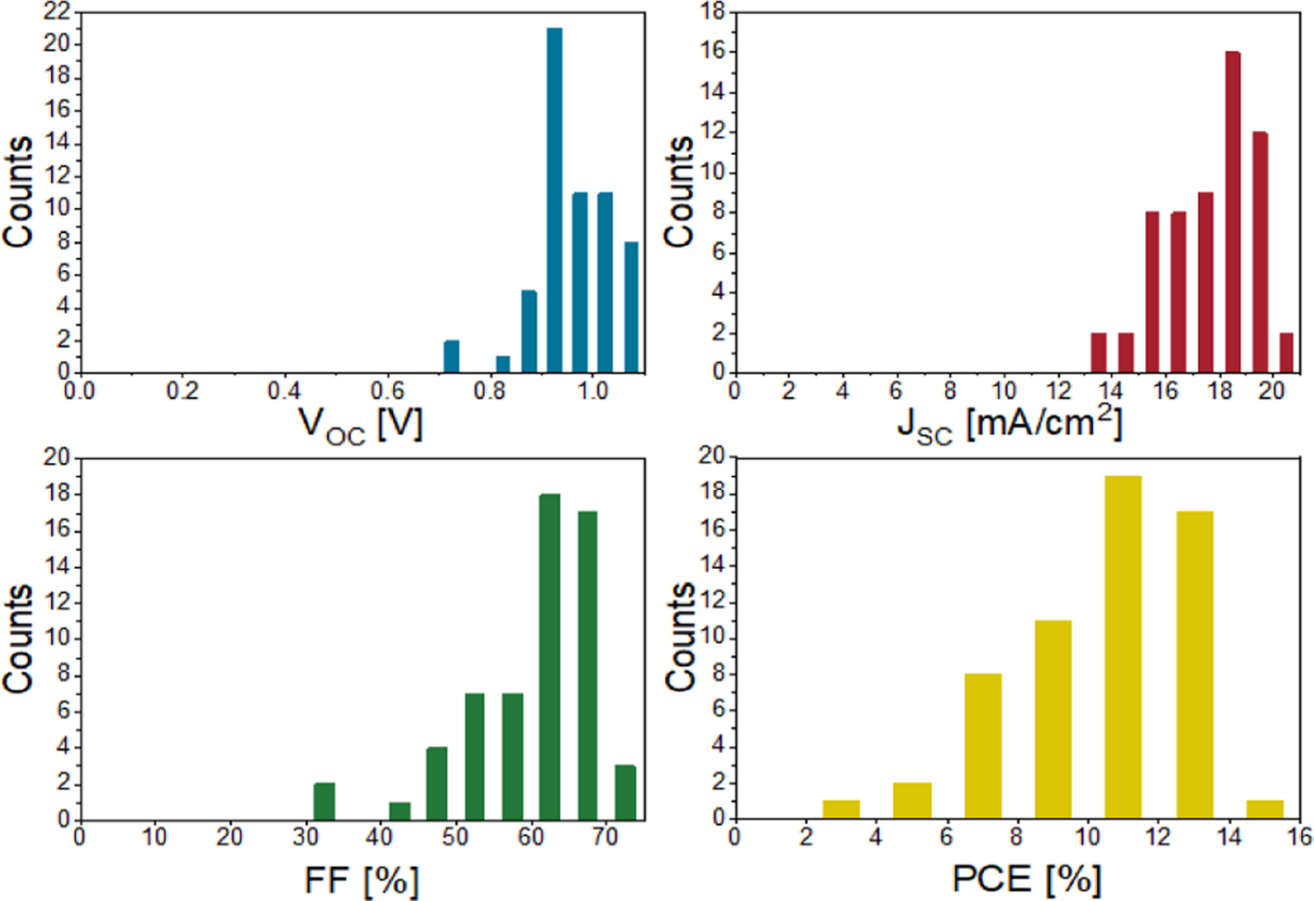
Statistical distribution of open-circuit voltage (*V*_OC_), current density (*J*_SC_), FF, and PCE of 59 flexible solar cells with PET/ITO/SnO_2_-NP/perovskite/spiro-OMeTAD/Au architecture from eight different batches fabricated over 6 months.

Figure 7 illustrates the statistical distribution of PV parameters measured for all 59 FPSCs that incorporated the SnO_2_-NP single ETL (from eight different batches fabricated over the course of 6 months of the study). The PCE distribution is mainly influenced by *J*_SC_ and FF distributions. Of the 59 cells, 51 devices give a high V_OC_ in the range from 0.9 to 1.1 V. The FF distribution is mostly found in the 60–70% range (60% of all cells). 5% of the cells reached an FF above 70%. Out of 59 cells, 19 had an efficiency of 10% or higher, 17 had an efficiency of 12% or higher, and the best cell with SnO_2_-NP single ETL reached a PCE of 14.1%.

Further improvements in the future may be achieved by various treatments that can improve charge extraction or diminish the density of the trap state in the perovskite layer and at the perovskite/ETL interface.^57,58^ A strategy that could help improve the stability of the ZnO/SnO_2_ cells could be that of increasing the thickness of the SnO_2_ layer, as reported in the case of Al_2_O_3_ layers over ZnO with an increasing thicknesses of Al_2_O_3_ films.^22^ Nevertheless, SnO_2_-NP films represent an advantageous and easy-to-manufacture ETL specifically for FPSCs combining good performance, efficiency, and ease of fabrication. Furthermore, a planar device with SnO_2_-only ETL means there is only one layer to be deposited, decreasing the production time and cost.^55,59,60^

### 3.2 Understanding What Makes a Good ETL for FPSCs in Comparison with Those on Glass

In order to study performance differences when shifting from glass to flexible substrates, keeping the same architectures, we carried out a new set of eight types of devices on glass and flexible substrates: that is, SnO_2_, SnO_2_/MgO, ZnO/SnO_2_, and SnO_2_/Al_2_O_3_.

#### 3.2.1 PV Performance of PSCs on Flexible PET versus Glass

The average PV parameters of all flexible and glass solar cells as a whole, as well as series resistance (*R*_S_), shunt resistance (*R*_SH_), forward and reverse currents under dark conditions (*I*_on_ and *I*_off_, respectively), and contact angles are summarized in Table 2 (see Table S2 for a breakdown for each type of ETL). As reported in the third column of Table 2, PCEs of glass cells were larger on average by 29% compared to cells fabricated on PET, with all PV parameters being boosted, especially *J*_SC_ (+15%), then FF (+7%), and *V*_OC_ (+6%).

**Table 2:** Average PV Parameters, Series Resistance *R*_S_, Shunt Resistance *R*_SH_, Forward Current *I*_on_, Reverse Current I_off_, Contact Angles, and Relative Variation of All Parameters between PSCs Fabricated on Glass Versus Those on PET^a^

	*V*_OC_ (V)	*J*_SC_ (mA/cm^2^)	FF (%)	PCE (%)	*R*_S_ (Ω)	*R*_SH_ (Ω)	*I*_on_ (mA)	*I*_off_ (mA)	contact angle (°)
PET cells	0.96 ± 0.06 [1.04]	15.7 ± 2.9 [17.1]	51.9 ± 3.2 [66.07]	7.9 ± 1.8 [11.75]	319 ± 33 [448]	6534 ± 3087.8 [8960]	0.044 ± 0.02 [0.09	2.6 × 10–^3^ ± 1.7 × 10–^3^ [1.3 × 10–^3^]	71.5 ± 8.7
glass cells	1.02 ± 0.05 [1.02]	18.1 ± 3.0 [18.72]	55.5 ± 3.4 [66.0]	10.2 ± 1.9 [12.6]	286 ± 43 [302]	11 405 ± 2172 [20 800]	0.047 ± 0.049 [0.131]	6.32 × 10–^4^ ± 2.34 × 10–^4^ [4.97 × 10–^4^]	53.5 ± 4.7
relative variation between glass and PET cells	+6.3% [+1.9%]	+15.6% [+9.5%]	+6.9% [+0.12%]	+29.4% [+7.3%]	–10.4% [−32.6%]	+74.6% [+132%]	+6.8% [+45.6%]	–75.7% [−62%]	–25.2%

a In brackets, we report the values for the best cells.

#### 3.2.2 How Shunt Resistances and Recombination Currents Underlie Performance Differences between Solar Cells

**Figure 8:**
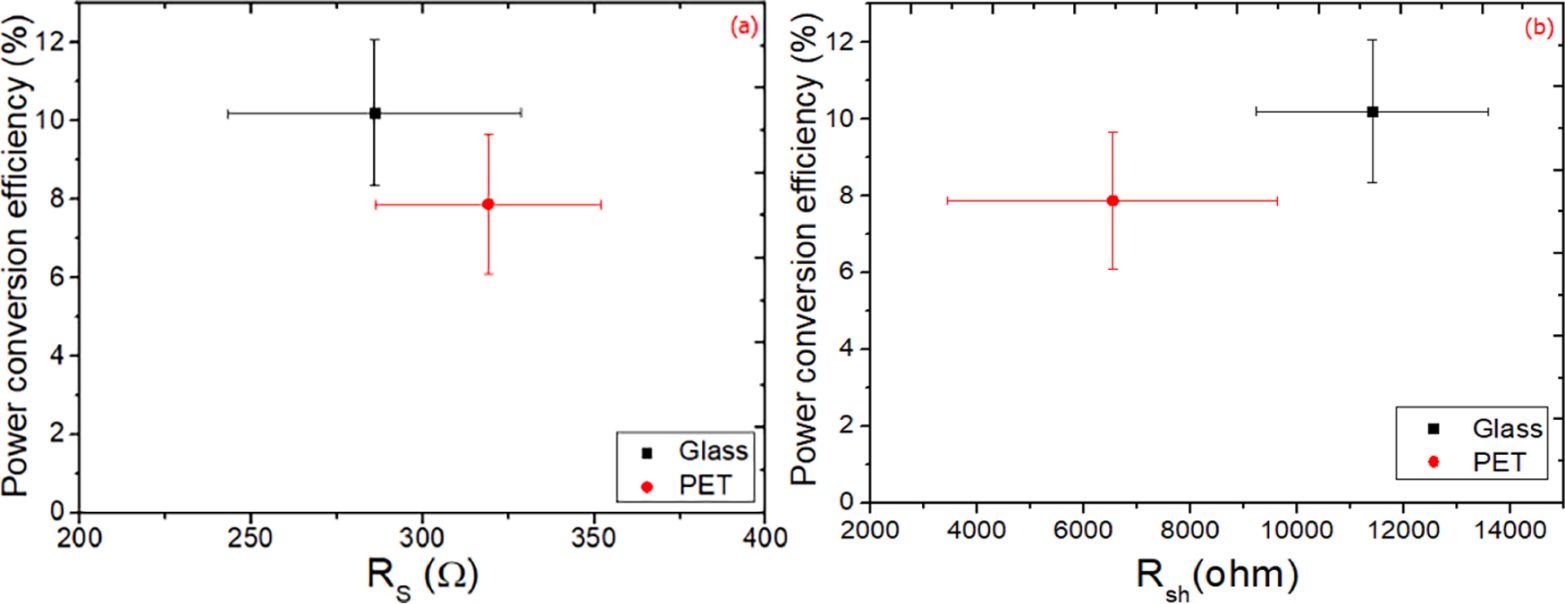
Dependence of average PCEs on series resistance (*R*_S_) (a) and shunt resistance (*R*_SH_) (b) of all flexible and rigid PSCs.

Figure 8 reports *R*_S_ and *R*_SH_ of all PET cells compared to those of all glass cells. It can be noticed that there is a decrease of around 10% in *R*_S_ for the latter. This is due to ITO being more conductive (*R*_sheet_ was 8 and 15 Ω/sq for glass and PET-based substrates, respectively) which does not however make a significant difference at the very small cell level but would rather at the module level with wide ITO strips,^58,61^ as well as glass cells possessing better layers/interfaces. Instead, *R*_SH_ doubles for glass-based devices versus PET, with the PCE increasing by 29% in relative terms, as a result mainly of higher *J*_SC_ and FF, as illustrated in Figure S5.

**Figure 9:**
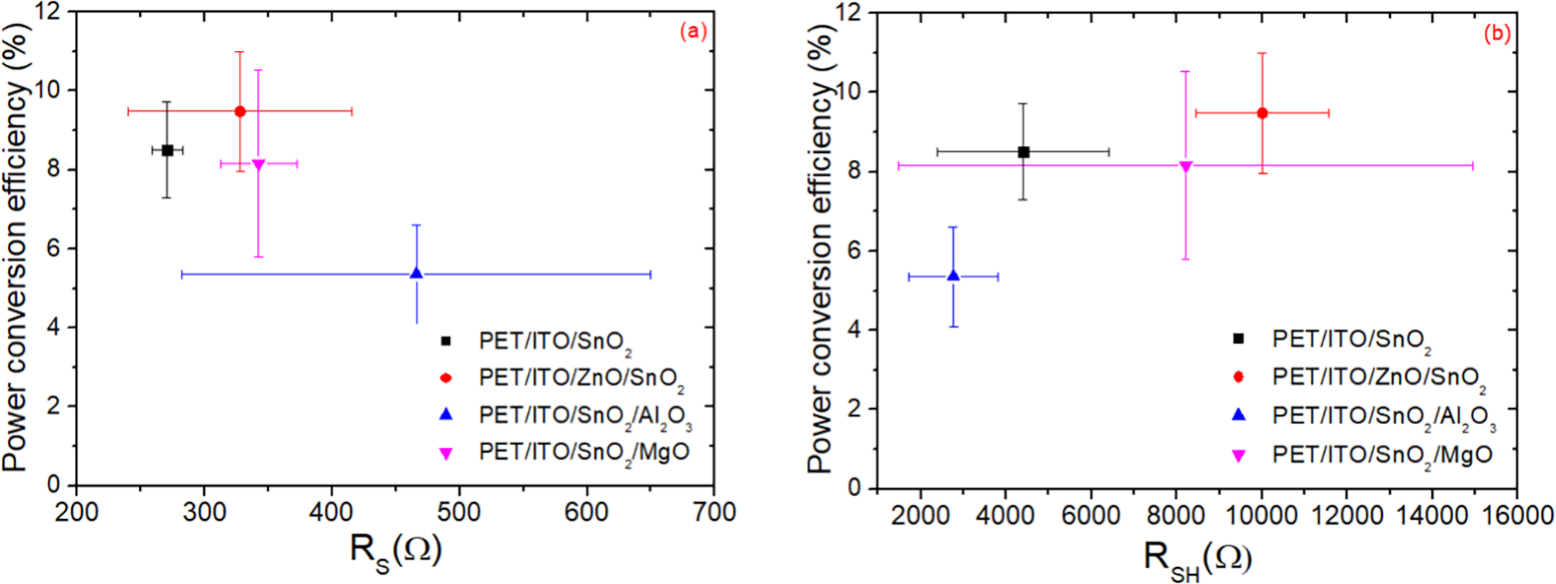
Dependence of average PCE on series resistance (*R*_S_) (a) and shunt resistance (*R*_SH_) (b) over the FPSCs with four different ETLs.

If we analyze the PET devices only (see Figure 9), the influence of the ETL on *R*_SH_ is indeed huge. *R*_SH_ was found to vary from roughly 2 × 10^3^ Ω for the cells with SnO_2_/Al_2_O_3_ bilayers to over 10 × 10^3^ Ω for the cells with ZnO/SnO_2_ bilayers. We found that when *R*_SH_ increased by a factor of ∼3, the average PCE of flexible cells almost doubled (from 5 to 9.5% in this batch of flexible cells studied). A good ETL needs to limit recombination losses, more so than improving *R*_S_. The variation of the latter is smaller but still significant in relative terms, that is, roughly a factor of 2, and can be mainly attributed to higher electron mobility, improved interface, improved perovskite morphology, and well-matched energy levels of ETL, which facilitate charge extraction from the perovskite to the electrodes.^62−65^ Lower shunt resistances, instead, generally arise from pinholes in the ETL as well as poor hole blocking behavior and interfacial defects, which produce recombination losses.^65−67^

**Figure 10:**
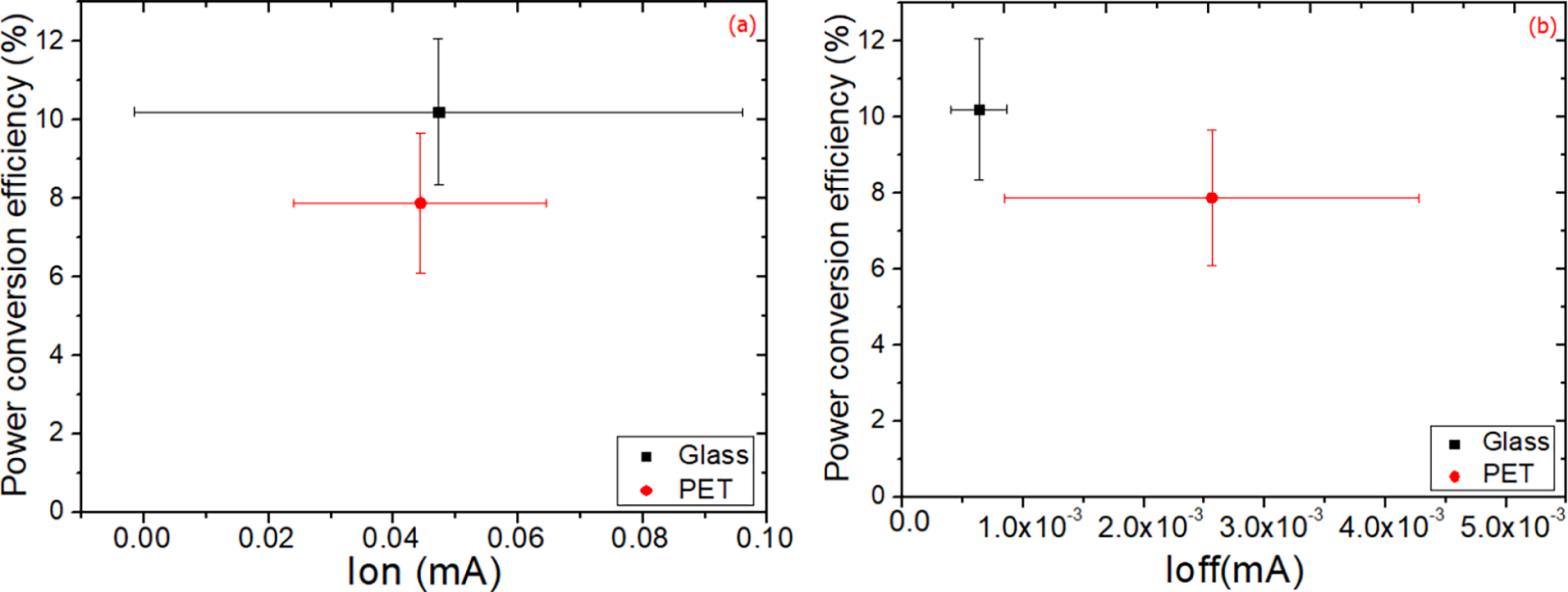
Average PCEs of all flexible and rigid devices as a function of forward (*I*_on_) (a) and reverse (*I*_off_) (b) currents measured in the dark extracted at 1 and −1 V, respectively.

To delve deeper in the differences, Figure 10 shows that devices fabricated on glass have slightly higher on-dark currents, *I*_on_, as well as significantly lower currents, *I*_off_. The former have a smaller impact on cell performance compared to the latter. In fact, the reverse bias current is 76% lower in relative terms on average for the glass devices, indicating considerably better blocking behavior, confirming the findings on shunt resistance. When the dark current is 76% lower, a relative 29% increase in PCE of glass devices was obtained. This leads to an improvement mainly in *J*_SC_ and FF, as shown in Figure S6b,c. The lower reverse dark currents in glass devices compared to the ones on PET is a result of better blocking behavior and lower recombination currents at the electrode/ETL/perovskite interfaces.^43,68^ Whereas a breakdown of performance versus resistances on the different devices on PET confirms semiquantitatively the importance and the effect of these resistances (Figure 9), it was not possible to find a quantitative trend in *I*_on_ and *I*_off_ due to the inherent large variation between devices for these parameters on PET (see Figure S7).

#### 3.2.3 Influence of Wetting and Contact Angle on Final Solar Cell Performance

**Figure 11:**
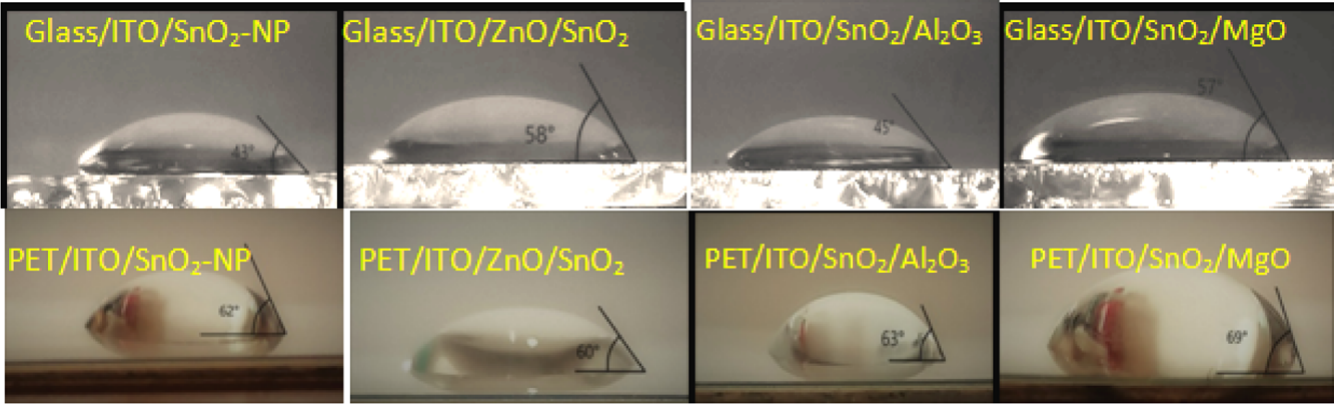
Water contact angle measurements over the different ETLs on glass (above) and PET (below) substrates.

**Figure 12:**
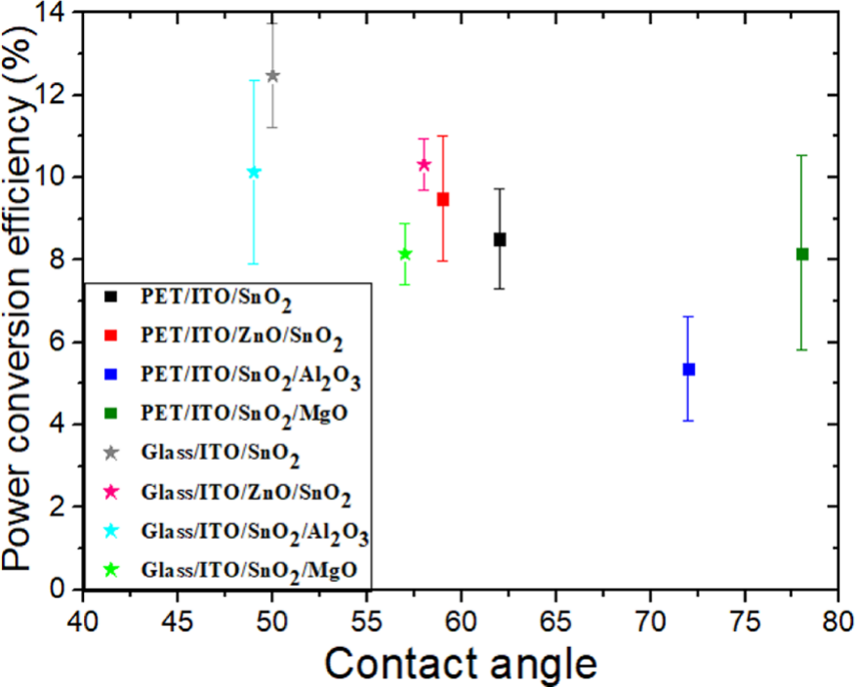
PCEs as a function of contact angles for both flexible and rigid PSCs fabricated with different ETLs.

In order to ascertain the origin of the better quality of the ETLs and the resulting better *R*_S_, *R*_SH_, *I*_off_, and PCE on glass substrates compared to those on PET, we performed contact angle measurements^69^ on the different ETLs on the different substrates (Figure 11) since it is the ITO/ETL/perovskite interface which determines to a great extent the series resistance and blocking behavior in the cells. The contact angle of the SnO_2_ layers increased from 49 to 77° when measured on glass and flexible substrates/ETLs, respectively (Figure 12). Better wetting at the interface ensures the formation of a perovskite film that is free of pinholes that would form shunt paths by inducing direct contact between the HTL and the ETL and reduce open-circuit voltage and FF. ETLs with better wetting properties can provide more complete coverage of the film with better crystallinity by assisting the nucleation of the perovskite and passivating crystal grain boundaries.^58,70^ The resulting higher-quality films translate in better PV performance, thus largely explaining the difference between PSCs on glass and flexible substrates.^6,69,71^Figure 12, in fact, permits us to highlight a more general trend: lower contact angles and better wetting of the inks over the electrode/ETL surface are conducive to delivering higher-quality transport layers, as well as interfaces with the perovskite, which in turn lead to better performance.^6,58,72,73^

## 4 Conclusions

We fabricated FPSCs using 12 different kinds of ETLs. Among all the ETLs, the cells with ZnO/SnO_2_ delivered a highest PCE of 14.8% at one sun illumination. The average PCE of ZnO/SnO_2_-based flexible PSCs was 39% higher, in relative terms, compared to SnO_2_-based cells fabricated in the same batch. With regard to the stability, the cells with SnO_2_-NP-only ETL were found to be more stable than cells with ZnO/SnO_2_ double ETL and generally more stable than all the double ETL-based devices investigated in this work. Thus, the most suitable of all 12 ETLs studied here was that consisting of SnO_2_ NPs. FPSCs with SnO_2_-NP delivered a PCE which was on average 250% higher than that of the same cells fabricated with SnO_2_ made with a liquid precursor.

Our study to understand the characteristics of what makes a good ETL on PET revealed that a good ETL should ensure high mobility, provide high shunt resistance, and reduce recombination currents. The main differences between glass and PET devices that give a performance advantage on glass (+29% in PCE in relative terms) are mainly found in shunt resistances and recombination currents. Moreover, we found that the wetting behavior of each ETL and the final performance of the device tended to be related for all ETLs and substrate types. Cell efficiency increases with a low contact angle, which is 25% lower for glass devices compared to flexible devices.

We can conclude that in order to obtain high-performing solar cells on PET/ITO substrates, it is necessary to focus and enhance more the blocking behavior of the ETL (rather than its transport properties). Furthermore, it is important to improve the wetting properties of the conductive substrate and ETL systems since achieving high-quality layers and interfaces was found to be related to better wetting of inks. These findings will be helpful for those laboratories migrating from glass devices to flexible ones, as well as those wanting to develop high-quality foundational ETLs over which device fabrication on plastic substrates starts.

## Supporting Information Available

PCE statistics, light stability, best PV parameters, average PV parameters, dependence of PV parameters on series resistance, shunt resistance, and reverse current (PDF)
