# Supplementary material for: Attributes of High-Performance Electron Transport Layers for Perovskite Solar Cells on Flexible PET versus on Glass
Source: ACS Appl Energy Mater. 2022 Apr 6;5(4):4096–107. doi: 10.1021/acsaem.1c03311 (PMC9044394; doi:10.1021/acsaem.1c03311)
Supplement: Supplementary file 1 — ae1c03311_si_001.pdf [file ae1c03311_si_001.pdf]

Supporting Information

Attributes of high-performance electron transport layers for perovskite solar cells on flexible PET vs on glass

Marwa Dkhili[a,b,c,d], Giulia Lucarelli[a], Francesca De Rossi[a], Babak Taheri[a], Khadija Hammedi[b,c,d], Hatem Ezzaouia[b,c], Francesca Brunetti[a], Thomas M. Brown[a*]*

[a] CHOSE (Centre for Hybrid and Organic Solar Energy), Department of Electronic Engineering, University of Rome Tor Vergata, Via del Politecnico 1, 00133 Rome, Italy
[b] Laboratory of Semiconductors, Nanostructures and Advanced Technology (LSNTA), Research and Technology Center of Energy (CRTEn), BP 95, 2050 Hammam-Lif, Tunisia
[c] Photovoltaic Laboratory, Research and Technology Centre of Energy (CRTEn), BP 95, 2050 Hammam-Lif, Tunisia
[d] Faculty of Sciences of Tunis, El Manar University, 2092 Tunis, Tunisia

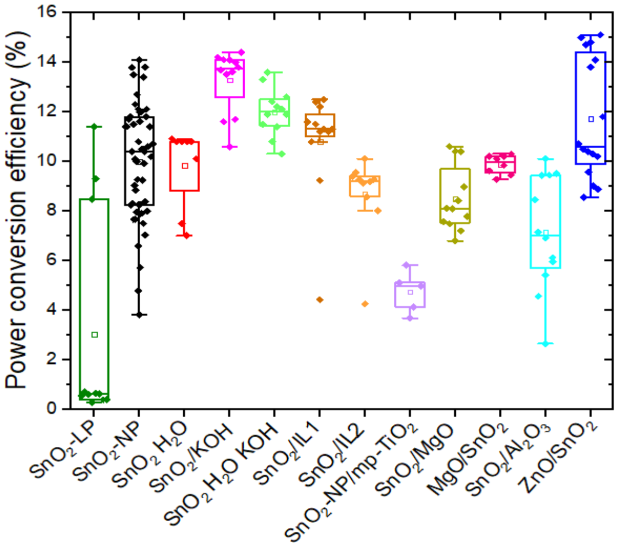

Figure S1. Statistical distribution of power conversion efficiency of flexible perovskite solar cells on PET/ITO substrates based on 12 different electron transport layers i.e. SnO_2-LP, SnO_2-NP, SnO_2-H_2O, SnO_2/KOH, SnO_2-H_2O/KOH, SnO_2/IL1, SnO_2/IL2, SnO_2/mp-TiO_2, SnO_2/MgO, MgO/SnO_2, SnO_2/Al_2O_3 and ZnO/SnO_2, where IL1 and IL2 are 1-benzyl-3-methylimidazolium chloride and 1-butyl-3-methylimidazolium tetrafluoroborate, respectively

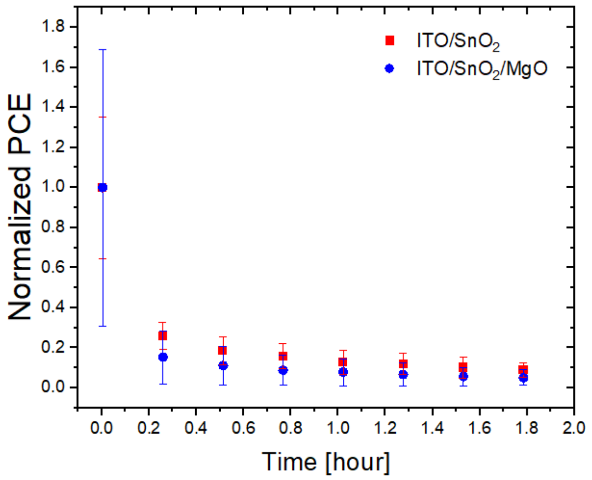

Figure S2. Light soaking stability measurements, at 1 sun illumination, under ambient temperature and relative humidity (R.H) in the range of [30, 60%], over time of PET/ITO/electron transport layer/perovskite/spiro-OMeTAD/Au architecture based on SnO_2 (Red squared data points) and SnO_2/MgO (Blue circles)electron transport layers.

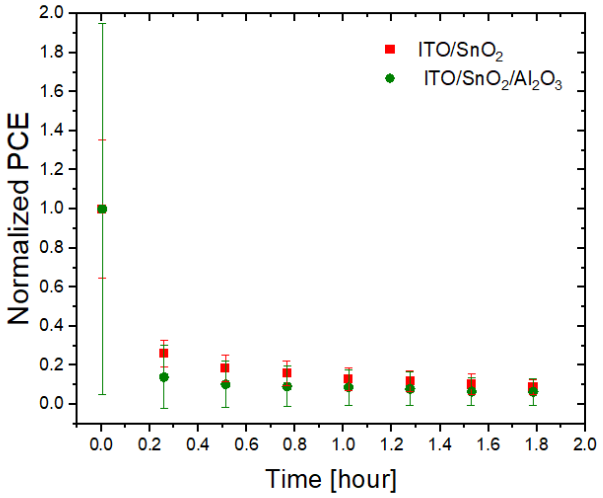

Figure S3. Light soaking stability measurements, at 1 sun illumination, under ambient temperature and relative humidity (R.H) in the range of [30, 60%], over time of PET/ITO/electron transport layer/perovskite/spiro-OMeTAD/Au architecture based on SnO_2 (Red squared data points) and SnO_2/Al_2O_3 (Green circles) electron transport layers.

Table S1. Power conversion efficiencies of the best cells measured for the different ETLs-based perovskite solar cells under standard test conditions.

Electron transport layers	*PCEs of the best cells (%)*
	Reverse scan
SnO_2-NP	*14.1*
SnO_2-LP	*11.4*
SnO_2-H2O	*10.9*
SnO_2/KOH	*11.9*
SnO_2-H_2O/KOH	*13.1*
SnO_2/IL1	*12.5*
SnO_2/IL2	*10.1*
SnO_2/meso-TiO_2	*8.94*
SnO_2/MgO	*10.6*
MgO/SnO_2	*10.3*
SnO_2/Al_2O_3	*10.1*
ZnO/SnO_2	*14.8*

Table S2. Average PV parameters with best cells of flexible and rigid perovskite solar cells with different ETL types.

**Device type**	**Voc (V)**	**Jsc (mA/cm²)**	**FF (%)**	**PCE(%)**
**SnO_2-PET**	0.94±0.02 [0.92]	18.39±0.08 [18.45]	49.2±5.77 [53.28]	8.51±1.22 [9.37]
**SnO_2-Glass**	0.95±0.08 [1.02]	21.86±3.003 [18.72]	60.41±4.84 [65.99]	12.47±1.27 [12.61]
**SnO_2/Al_2O_3-PET**	0.9±0.04 [0.94]	11.85±1.5 [13.48]	49.69±3.22 [53.41]	5.36±1.25 [6.79]
**SnO_2/Al_2O_3-Glass**	1.01±0.01 [1.02]	18.37±1.66 [19.22]	54.08±7.73 [62.85]	10.13±2.21 [12.36]
**SnO_2/MgO-PET**	0.95±0.14 [1.05]	15.22±2.26 [17.05]	56.09±8.11 [64.93]	8.17±2.36 [11.61]
**SnO_2/MgO-Glass**	1.03±0.03 [1.06]	14.55±1.11 [13.27]	52.89±5.11 [56.78]	7.89±0.65 [7.99]
**ZnO/SnO_2-PET**	1.05±0.01 [1.04]	17.23±0.31 [17.1]	52.5±9.1 [66.07]	9.49±1.51 [11.75]

**ZnO/SnO₂-Glass**	1.07±0.02 [1.08]	17.67±0.33 [17.90]	54.43±1.44 [55.45]	10.32±0.62 [10.76]

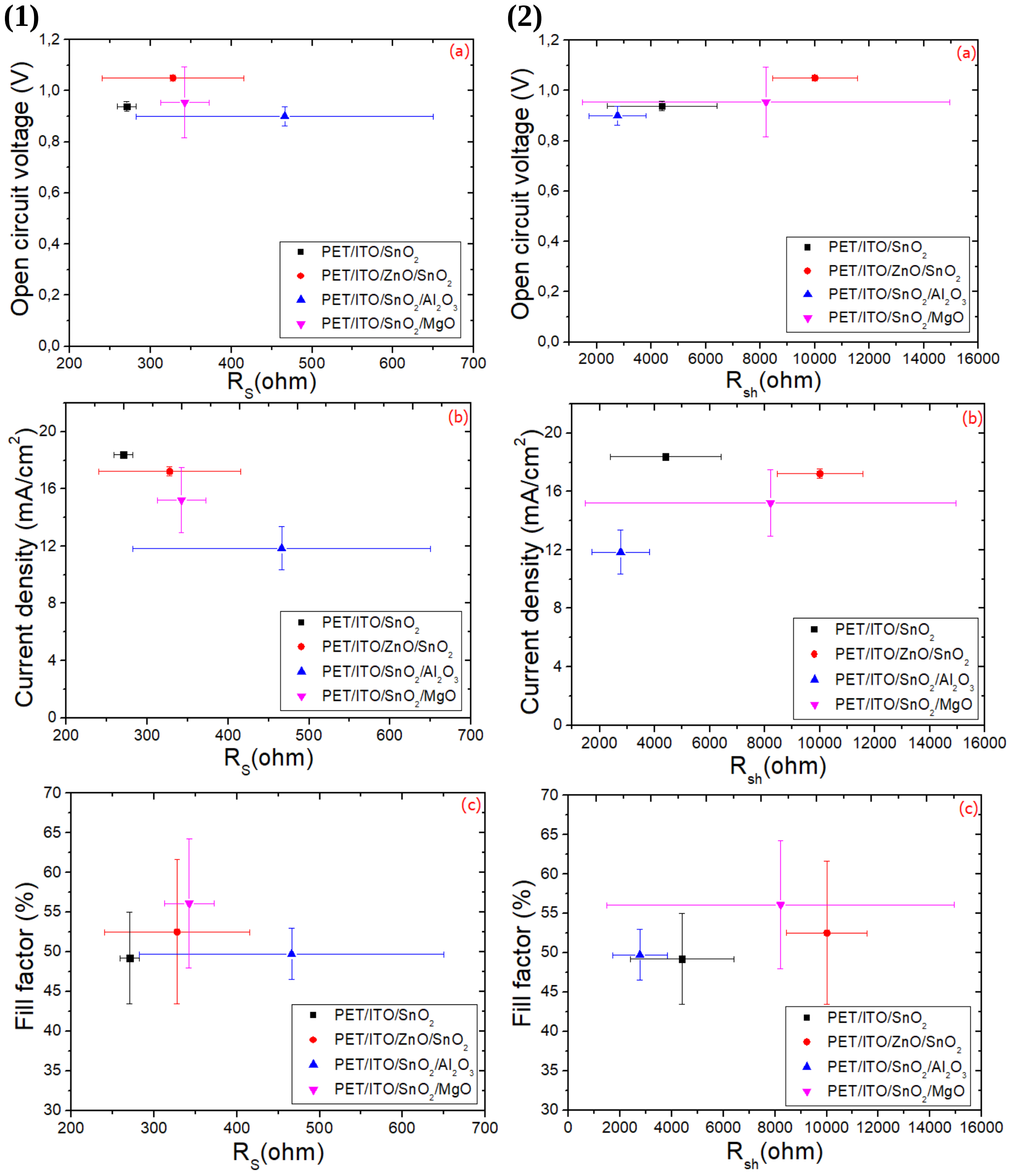

Figure S4. Dependence of average V_{oc} (a), J_{sc}(b) and FF(c) on series resistance (1) and shunt resistance (2) of four electron transport layers-based flexible perovskite solar cells.

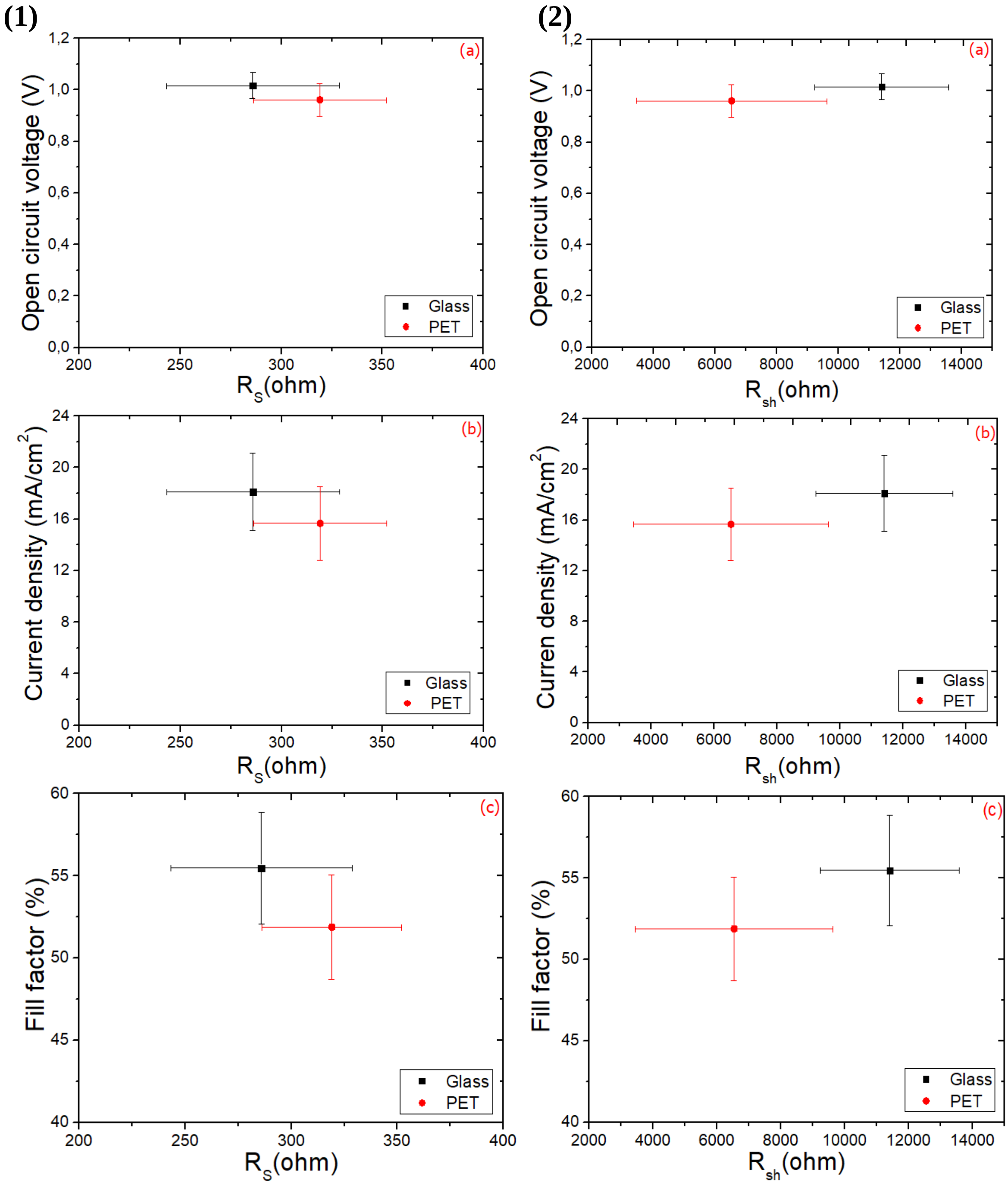

Figure S5. Dependence of average V_{oc} (a), J_{sc}(b) and FF(c) on series resistance (1) and shunt resistance (2) of all flexible and glass perovskite solar cells.

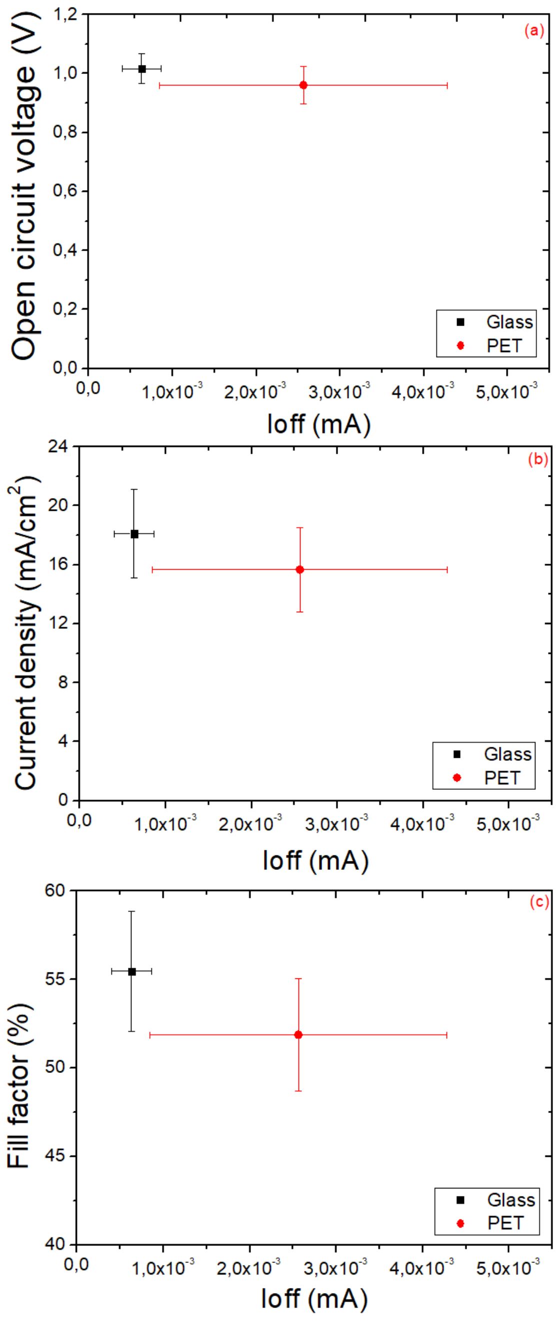

Figure S6. Average V_{oc} (a), J_{sc} (b) and FF (c) of all flexible and rigid devices based on different ETLs as a function of reverse currents.

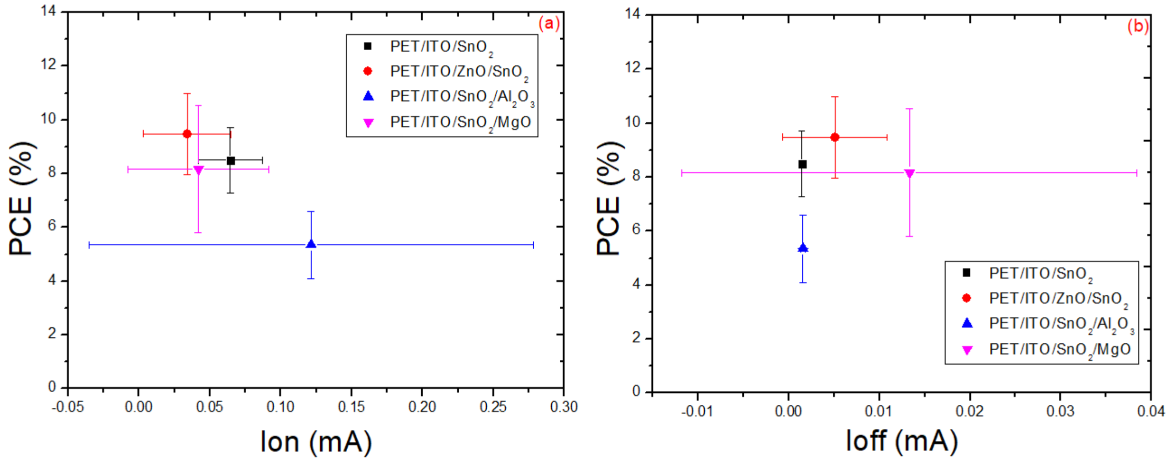

Figure S7. Average power conversion efficiencies (PCE) of four electron transport layers-based flexible perovskite solar cells as a function of forward (a) and reverse (b) currents measured in the dark extracted at 1V and -1 V, respectively.
